# Biomarkers in previous histologically negative prostate biopsies can be helpful in repeat biopsy decision‐making processes

**DOI:** 10.1002/cam4.3419

**Published:** 2020-08-28

**Authors:** Xingbo Long, Longxiang Wu, Xiting Zeng, Zhijian Wu, Xiheng Hu, Huichuan Jiang, Zhengtong Lv, Changzhao Yang, Yi Cai, Keda Yang, Yuan Li

**Affiliations:** ^1^ Department of Urology Beijing Hospital National Center of Gerontology Beijing China; ^2^ Graduate School of Chinese Academy of Medical Science and Peking Union Medical College Beijing China; ^3^ Department of Urology Xiangya Hospital Central South University Changsha China; ^4^ Department of Ophthalmology The Affiliated XiangTan Hospital XiangYa Medical College CSU (XiangTan Central Hospital) XiangTan Hunan China; ^5^ Department of Urology Chenzhou No.1 People’s Hospital ChengZhou Hunan China; ^6^ Department of Pathology Xiangya Hospital Central South University Changsha Hunan China

**Keywords:** Biomarker, Decision‐making process, Field effect, Prostate cancer, Repeat prostate biopsy

## Abstract

To evaluate whether the addition of biomarkers to traditional clinicopathological parameters may help to increase the accurate prediction of prostate re‐biopsy outcome. A training cohort with 98 patients and a validation cohort with 72 patients were retrospectively recruited into our study. Immunohistochemical analysis was used to evaluate the immunoreactivity of a group of biomarkers in the initial negative biopsy normal‐looking tissues of the training and validation cohorts. p‐STAT3, Mcm2, and/or MSR1 were selected out of 10 biomarkers to construct a biomarker index for predicting cancer and high‐grade prostate cancer (HGPCa) in the training cohort based on the stepwise logistic regression analysis; these biomarkers were then validated in the validation cohort. In the training cohort study, we found that the biomarker index was independently associated with the re‐biopsy outcomes of cancer and HGPCa. Moreover supplementing the biomarker index with traditional clinical‐pathological parameters can improve the area under the receiver operating characteristic curve of the model from 0.722 to 0.842 and from 0.735 to 0.842, respectively, for predicting cancer and HGPCa at re‐biopsy. In the decision‐making analysis, we found the model supplemented with the biomarker index can improve patients’ net benefit. The application of the model to clinical practice, at a 10% risk threshold, would reduce the number of biopsies by 34.7% while delaying the diagnosis of 7.8% cancers and would reduce the number of biopsies by 73.5% while delaying the diagnosis of 17.8% HGPCas. Taken together, supplementing the biomarker index with clinicopathological parameters may help urologists in re‐biopsy decision‐making processes.

## Graphical abstract

p‐STAT3, Mcm2, and/or MSR1 were selected out of 10 biomarkers to construct biomarker index. Biomarker index are associated with positive repeat prostate biopsy results. Supplementing biomarker index help urologists in re‐biopsy decision‐making processes.

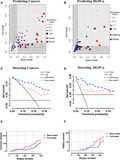

## 1 INTRODUCTION

Prostate cancer (PCa) is one of the most frequently diagnosed malignancies among men worldwide.^1^ Currently, prostate biopsy is regarded as the gold standard in the evaluation of abnormal PCa screening results. However, because of the 20 to 40% false‐negative rate of the initial biopsy and persistent abnormal clinical‐pathological parameters more than 50% of patients who undergo initial biopsy are left with doubt regarding the potential presence of PCa and need repeat biopsy after a period of follow‐up.^2^, ^3^, ^4^, ^5^ Despite the numbers of traditional clinical‐pathological parameters, including prostate‐specific antigen (PSA), percentage of free‐PSA (fPSA), PSA velocity, and presence of high‐grade prostatic intraepithelial neoplasia (HGPIN) or atypical small acinar proliferation (ASAP) in the previous negative biopsy tissues were used to screen patients for repeat biopsy, unfortunately, the positive biopsy rate among men undergoing repeat biopsy is only approximately 20% to 40% and high grade PCa (HGPCa) accounted for less than half, which suggests that three out of every five biopsies are unnecessary.^2^, ^3^, ^4^, ^5^, ^6^ Prostate biopsy can be painful, anxiety‐provoking, expensive, and potentially morbid, so there is an urgent need to supplement the above mentioned traditional parameters with novel biomarkers that enhance its specificity so that unnecessary biopsies can be avoided.

Field effect (Also known as the field cancerization) refers to genetically altered but phenotypically normal‐looking tissues surrounding a cancer focus.^7^, ^8^ Increasing evidence has shown that the field effect may also persist in PCa.^9^, ^10^, ^11^, ^12^ Prostatic tissue adjacent to tumors may be influenced by the tumor tissues and different from more remote prostatic tissue. Inflammatory cellular infiltration, abnormal angiogenesis, stromal structural changes, and dysregulated gene and protein expression would be observed in this field.^7^, ^13^ Such changes may provide valuable information for the potentially undetected tumors. Therefore, this type of tissue could be proposed to be termed phenotypically normal‐looking tumor tissue (PNTI). For patients who actually have PCa but are negative at the initial biopsy, the initial biopsy might cover PNTI areas but not reach the actual tumor. Therefore, PNTI in the initial biopsy may be useful to diagnose the presence and evaluate the aggressiveness of PCa in patients with repeat biopsy.

Some immunohistochemical studies have demonstrated a group of biomarkers which may have potential field effects and may be helpful in the repeat biopsy decision‐making processes. Those biomarkers including inflammatory and immune cell infiltration—[CD3 + T cells, macrophage scavenger receptor 1 (MSR1)+ cells and CD 68 + macrophages cells], angiogenesis—[CD31 + vascular endothelial cells and vascular endothelial growth factor (VEGF)], proliferation and apoptotic markers—[Ki‐67, minichromosome maintenance complex protein 2 (Mcm2) and active‐casp3 (a‐casp3)] and some protein expression markers—[p‐AKT and p‐STAT3].^11^, ^14^, ^15^, ^16^, ^17^, ^18^, ^19^

However, no studies have evaluated the joint performance of the above 10 biomarkers in a repeat biopsy cohort. Therefore, we designed the present study to investigate if above biomarkers are associated with repeat biopsy outcomes and if the combined biomarkers of various implications could enhance the capacity of decision‐making for repeat prostate biopsy.

## 2 MATERIALS AND METHODS

### 2.1 Patients and specimens

**Figure 1:**
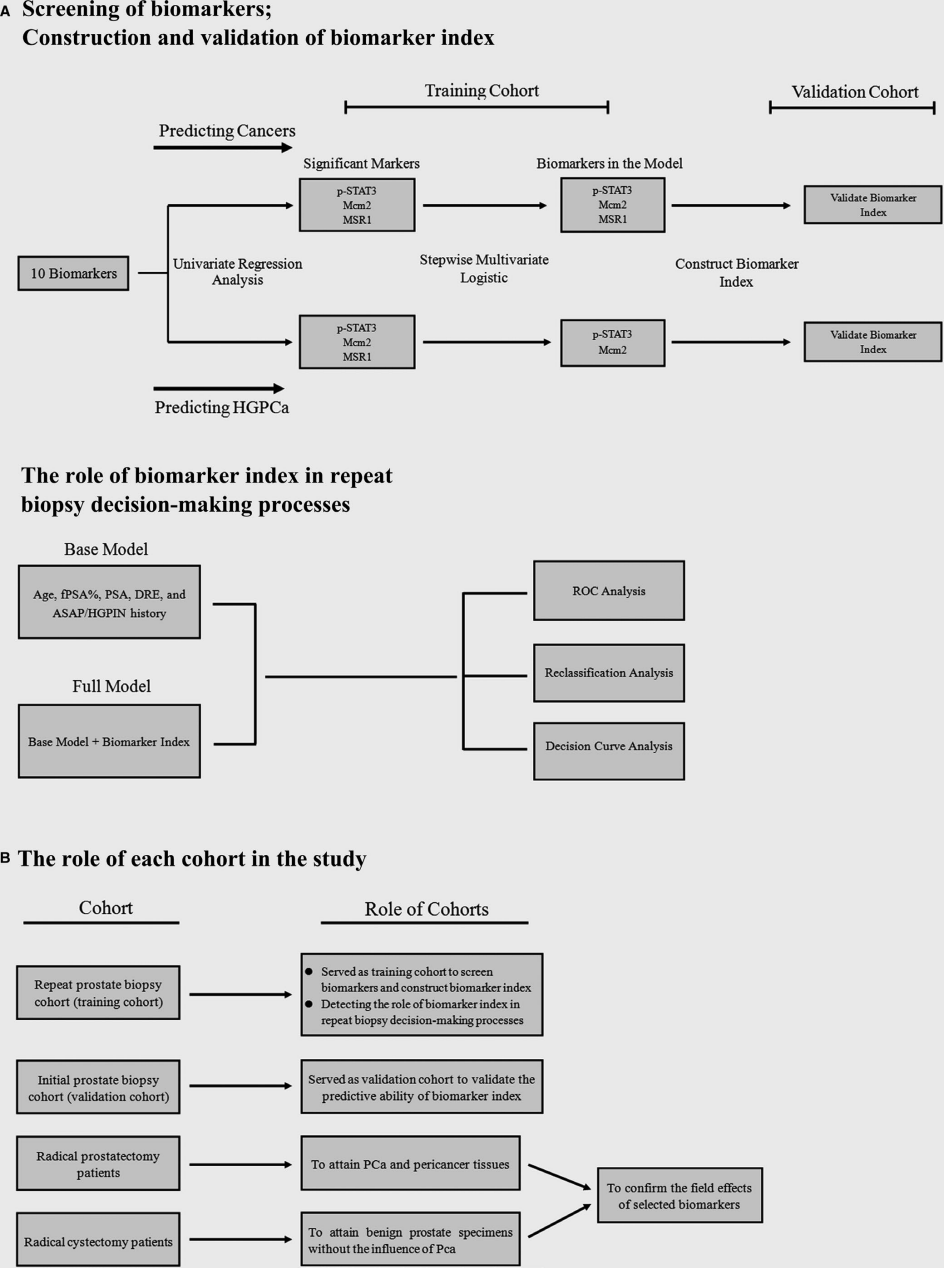
Study design and role of each cohort. (A) Study design. Briefly 10 biomarkers which proved may have potential field effects by previous studies and may be helpful in the repeat biopsy decision‐making processes were selected in our study. In the training cohort, univariate logistic analysis was used to screen above 10 biomarkers significantly associated with positive repeat biopsy results (cancer and HGPCa). And multivariate stepwise logistic regression modeling was employed to construct a model using all factors that were significant in the univariate analysis (*P* < .1). The prediction probability of the model multiplied by 100 was set as biomarker index. Then the predictive ability of biomarker index was validated in the validation cohort. Finally the role of biomarker index in repeat biopsy decision‐making processes was further validated by multivariate regression, ROC, reclassification and DCA analysis in the training cohort. (B) The role of each cohort in the study

The study design and role of each cohort was shown in Figure 1A and B respectively. This study was approved by the Ethics Committee at Xiangya Hospital, Central South University and Chenzhou NO.1 People's Hospital.

#### 2.1.1 Repeat prostate biopsy cohort (training cohort)

From the 2009 to 2016, after receiving approval from the Hospitals’ Ethics Committees, Xiangya Hospital of Central South University and Chenzhou NO.1 Hospital retrospectively provided data on the clinical and histopathological records of 98 consecutive patients with more than or equal to twice the prostate biopsies but were negative for the previous biopsy. The indications for repeat biopsy included persistently abnormal PSA or percentage of fPSA, presence of ASAP/HGPIN on an initial biopsy or new suspicious digital rectal exam (DRE) during follow‐up. To exclude extreme values, patients with serum PSA > 50 ng/ml, free/total PSA ratio > 0.5, and aged older than 90 years or less than 40 years were excluded from the study. The end points of follow‐up included: (a) patients were diagnosed with a PCa within 36 months or (b) at least 36 months of follow‐up were completed without finding PCa. Patients would also be excluded if they did not reach the end points of follow‐up. The clinical variables obtained from each patient during their initial biopsy process included age, serum PSA, percentage of fPSA and DRE findings. The presence of HGPIN and/or ASAP from the initial biopsy was diagnosed according to previously published criteria.^20^, ^21^ To minimize external influences, included patients were free of urinary retention, urinary tract infections, catheterization, and other transurethral operations during the 2 weeks prior to the serum PSA test. Patients did not receive 5α‐reductase inhibitors within the last two months. Previous biopsy samples of recruited patients were retrieved from Xiangya and Chenzhou NO.1 Hospital pathology repository. The repeat prostate biopsy cohort was used as the training cohort to construct the biomarker index, and the clinical efficiency of the biomarker index in repeat biopsy was also evaluated in this cohort. The clinical characteristics of patients are summarized in Table 1.

**Table 1:** Clinicopathological characteristics of the participants stratified by repeat biopsy results in the training cohort

Variables	Total (n = 98)	Repeat Biopsy Results			*P* Value	
		Benign (n = 73)	Any prostate cancer (n = 25)	HGPCa (n = 11)	Any cancer vs Benign	HGPCa vs Benign
Age,yr, (mean ± SD)	65.42 ± 7.19	65.16 ± 7.61	66.16 ± 5.84	64.55 ± 4.34	.55	.79
f/t PSA ratio, %, (mean ± SD)	16.55 ± 4.94	17.15 ± 4.75	14.80 ± 5.16	13.82 ± 5.72	.039	.038
Clinical serum PSA, ng/mL, (mean ± SD)	8.30 ± 4.52	7.74 ± 3.79	9.92 ± 5.99	10.88 ± 6.39	.037	.023
No. of suspicious DRE, n, (%)	7 (7.14)	5 (6.85)	2 (8.00)	1 (9.09)	1.00	.58
ASAP history, n, (%)	13 (13.27)	6 (8.22)	7 (28.00)	2 (18.18)	.19	.28
HGPIN history, n, (%)	13 (13.27)	9 (12.33)	4 (16.00)	1 (9.09)	.73	1.00
No. of previous biopsy cores (mean ± SD)	7.01 ± 1.85	6.9 ± 1.88	7.1 ± 1.79	7.55 ± 1.37	.73	.31

HGPCa, High grade prostate cancer

#### 2.1.2 Initial prostate biopsy cohort (validation cohort)

From 2014 to 2015, 72 consecutive patients from Xiangya and Chenzhou NO.1 Hospital who underwent initial biopsy because of suspicious of PCa were retrospectively recruited in our study. Among these patients, 24 patients were diagnosed with PCa during the initial biopsy session. The remaining 48 patients were not involved in further repeat biopsy sessions and were free of PCa during the 3 years of follow‐up after initial negative biopsy. The initial negative biopsy cores of these patients were collected for subsequent immunohistochemistry analysis. The initial prostate biopsy cohort served as an internal validation cohort to validate the predictive value of the biomarker index. The clinicopathological features of patients are summarized in Table S1.

#### 2.1.3 Source of PCa and pericancer tissues (Radical prostatectomy patients)

Sixteen specimens from radical prostatectomy due to pT2 PCa, which demonstrated a significant proportion of both neoplastic and benign tissues, were collected from Xiangya Hospital. Immediately after the procedure, the specimen was internally perfused and then externally perfused in 10% zinc formalin overnight. After fixation, the prostate gland was transversely sectioned at 0.3‐cm intervals from the apex to the base, in each case using the modification of the Stanford technique.^22^ An average of twenty blocks of prostatic parenchyma were obtained from each case. The block with significant proportions of both neoplastic and benign tissues was selected to attain pericancer tissues at different distances (5 mm and 10 mm) from the tumor using a 1‐mm grid. Both the tumor and pericancer tissues were collected for subsequent immunohistochemistry analysis. The clinicopathological features of patients are summarized in Table S2.

#### 2.1.4 Source of benign prostate specimens (Radical cystectomy patients)

To obtain prostate specimens without the influence of PCa, benign prostate specimens were collected from 16 patients with muscle‐invasive bladder cancer who underwent radical cystectomy. To minimalize the influence of bladder cancer, patients with stage T3 or higher stage and prostatic urethra tumor invasion were excluded from the study. All of those specimens were found to have same degrees of benign hyperplasia.

The patients were clinically free of other systemic therapy or infections before the study. The clinicopathological features of patients are summarized in Table S3.

### 2.2 Pathological analysis

B ultrasound‐guided transrectal cores targeted biopsy was performed according to our institution's protocol. Average biopsy cores in repeat and initial prostate biopsy cohort were summarized in Table 1 and Table S1. We labeled the biopsy samples according to the gland region, then we fixed them in separate test tubes with formaldehyde for subsequent pathological and immunohistochemical analyses. Histological analysis of those tissue slices was conducted by an experienced pathologist using a standard method described by previous studies.^23^ Standard Gleason scoring was used to evaluate the grade of the tumor. We defined HGPCa as any grade group (GG) ≥ 2.^24^

### 2.3 Immunohistochemistry and evaluation of immunostaining

Antibodies against CD3, CD68, MSR1, CD31, VEGF, p‐AKT, p‐STAT3, Ki‐67, Mcm2, and a‐casp3 were used to identify corresponding protein immunoreactivity.

Briefly, after incubation in 4% neutral buffer, para‐formaldehyde was used to fix the sections of specimens followed by embedding the specimens in paraffin; the fixed samples were then cut into 4‐5 μm slices. Before incubation with the corresponding primary antibodies and biotinylated secondary antibodies (Vector, Burlingame, CA), these sections were deparaffinized, hydrated, and antigen retrieved. The sources of antibodies and their dilutions are summarized in Table S4.

The slides were evaluated by observers who were blinded to the origin of the samples using an inverted Olympus microscope with the assistance of image processing software: Image Pro Plus 6.0 software (Media Cybernetics Inc). According to the staining proteins and previous studies,^11^, ^14^, ^15^, ^16^, ^17^, ^18^, ^19^ three methods were used to evaluate the immunostaining: (a) When scoring the immunoreactivity of VEGF, p‐STAT3, p‐AKT, and a‐casp3, we employed H‐score a semi‐continuous variable scoring system which has been used in large number of previous studies.^25^, ^26^, ^27^ In detail, the percentage of immunostaining and the staining intensity were recorded. The percentage of immunostaining was measure with the assistant of Image Pro Plus 6.0 software (Media Cybernetics Inc). The percentage of positive cells in each core was scored between 0% and 100%. For staining intensity, strongly, moderate, and weak intensity was defined as dark brown, tan, and light yellow staining areas and no staining was defined as the same staining as negative control. When grading the staining intensity, we use the optical density value generated by Image Pro Plus as an important reference. The represented pictures of different grades and their corresponding optical density value were showed in Figure S1A. H‐score was calculated using the following formula: (percentage of cells of weak intensity × 1) + (percentage of cells of moderate intensity × 2) + (percentage of cells of strong intensity × 3). As a consequence, H‐score provided a semi‐continuous score between 0 and 300 for each core.^25^, ^26^, ^27^ During the scoring process, we ignored stromal staining as previous studies do. (b) The CD31^+^ blood vessels and CD3, CD68, and MSR1 positive individual infiltrating cells were quantified as the numbers of cells or blood vessels per unit area of the entire sections and changed transformed into density as cells or blood vessels/mm^2^. (c) When scoring Ki‐67 and Mcm2, the luminal to basal ratio of the percentage of positive cells was calculated according to the previous study.^11^ Because tumor tissues lacked basal cells, we defined the Ki‐67 and Mcm2 scores in the tumor tissues as positive infinity.

Immunoreactivity was independently assessed by two investigators (YKD and WLX) who were blinded to the clinicopathological data. The mean value of scores assessed by these investigators was taken as the final result. If their scores differed widely, the values were discussed until an agreement was reached. Pearson correlation analysis was used to evaluate the inter‐rater reliability between two investigators. The result showed that biomarkers’ immunoreactivity scores were highly correlated between two investigators (R = 0.87, *P* < .001).

### 2.4 Statistical methods

Mann‐Whitney U statistics was used to compare the immunoreactivity of biomarkers between different groups. When screening biomarkers and constructing biomarker index, Univariate logistic analysis was used to screen biomarkers significantly associated with positive repeat biopsy results (cancer and HGPCa). Then multivariate analysis was performed by stepwise logistic regression modeling to construct a model using factors that were significant in the univariate analysis (*P* < .1) in the training cohort. Biomarker index was defended as the prediction probability of the model multiplied by 100. In detail biomarker index was set as 100 * 1/(e^‐P^ + 1). e is natural logarithm and *P* = β_0_ + β_1_ × X_1_ + β_2_ × X_2_ + β_3_ × X_3_ + …+ β_n_ × X_n_. X is the variable in the model, β_0_ is constant in equation. β_x_ is coefficient of the corresponding variable. Then the biomarker index was validated in the validation cohort. We stratified patients with high and low risk group based on the biomarker index. Patients’ biomarker index values higher than 30 and 14 were classified as high‐risk respectively, when predicting cancers and HGPCas. We chose the threshold value according to the LOESS smooth curves as previous studies do.^28^ The corresponding biomarker index value of the inflection region where the slope of the Loess curve goes from relatively stable to significantly rising is selected as our cut‐off value. In the training cohort, univariate and multivariate logistic regression models were used to test the significance of the association of each clinical variable and biomarker index with a positive repeat biopsy outcome (cancer or HGPCa) and to develop predictive models using the enter strategy. Area under the receiver operating characteristic curve (AUC) was used to assess how well each variable or model discriminated between patients with and without cancer or HGPCa. For internal validation, models were subjected to 1000 bootstrap resamples, and the calibration plot was used to illustrate the level of agreement between the model predictions and the true risk of finding cancer or HGPCa. Finally, reclassification and decision curve analyses were used to determine the clinical value of different models.^29^ Where categorized, According to the LOESS curve, model scores of 0.2 and 0.11 were used as threshold values to classify patients as high and low‐risk groups when predicting cancers and HGPCas. The data were analyzed using R software version 3.6.1 (https://www.R‐project.org/), MedCalc software (MedCalc software bvba, Ostend, Flanders, Belgium) and SPSS version 21 (SPSS Inc, Chicago, IL). For all tests, P‐values less than 0.05 were considered significant.

## 3 RESULTS

### 3.1 Screening of biomarkers and construction of biomarker index

**Figure 2:**
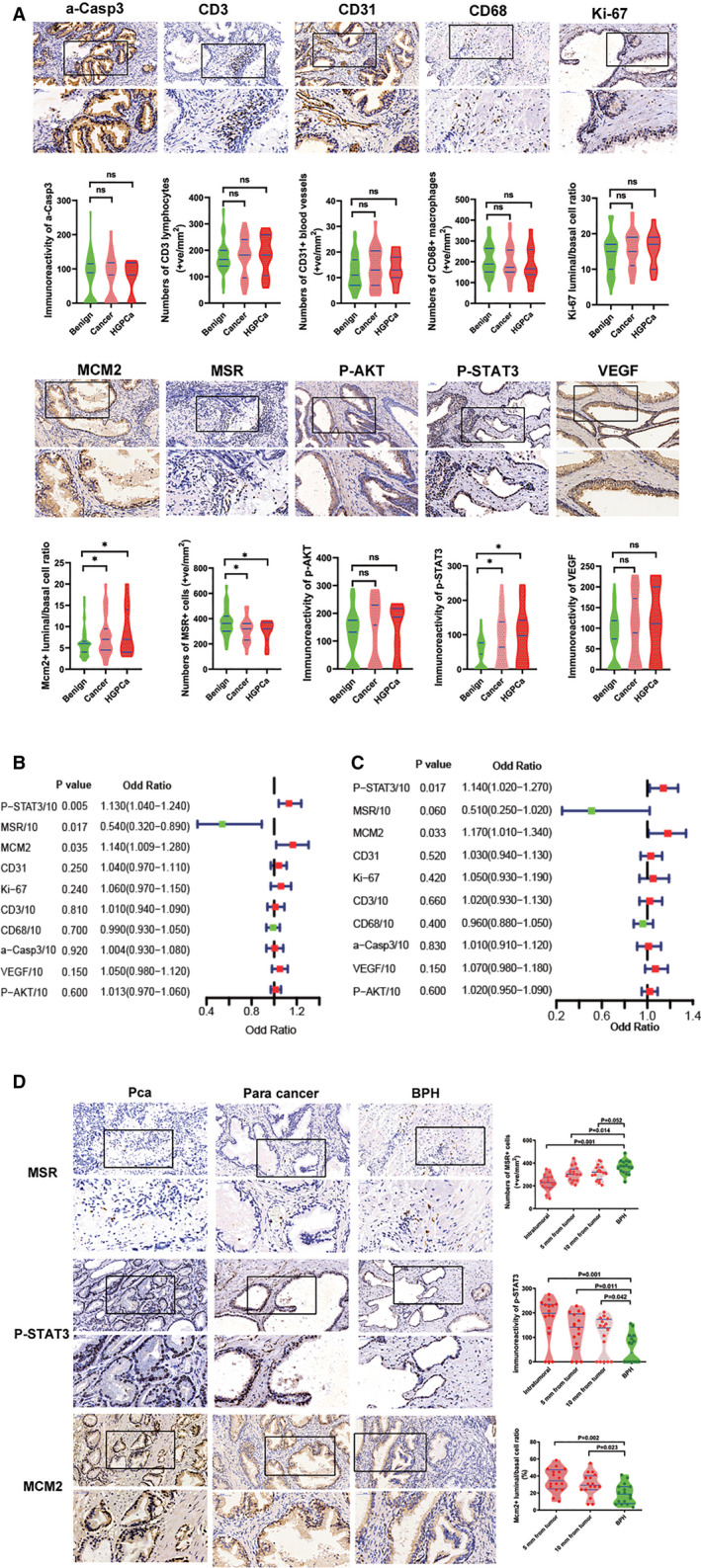
Screening of biomarkers and construction of biomarker index. (A) Immunoreactivity of biomarkers in initial negative biopsy samples of repeat biopsy cohort (original magnification x 20 and 40). Violin‐plot showed immunoreactivity of biomarkers in initial negative biopsy samples grouped by repeat biopsy result. Blue lines represent the median and the 25th to 75th percentiles. (B, C) Univariate logistic regression analysis of the 10 biomarkers when predicting cancer (B) and HGPCa (C) at repeat biopsy. (D) The immunoreactivity of p‐STAT3, Mcm2 + luminal to basal ratio and number of MSR + cells in PCa, pericancer tissues with different distances from the tumor area and benign prostate tissues (original magnification x 20 and 40). Because the prostate tumor tissues lacked basal cells, we defined the Mcm2 + luminal to basal ratio score in the tumor tissues as positive infinity and did not show it in the violin plot for the PCa group. Violin‐plot on the right showed immunoreactivity of biomarkers. Blue lines represent the median and the 25th to 75th percentiles

The immunoreactivity of biomarkers in the initial negative biopsy samples of the training cohort are shown in Figure 2A and Table S5. We found that the Immunoreactivity of p‐STAT3 and ratios of the luminal to basal of Mcm2 + cells in the initial negative biopsy tissues were significantly elevated, while the number of MSR1 + cells was significantly lower in those patients who were diagnosed with PCa and HGPCa as compared to those with a benign prostate biopsy (Figure 2A and Table S5). Univariate logistic regression analysis further confirmed that the immunoreactivity of p‐STAT3, Mcm2 + luminal to basal ratio and MSR1 + cell number in the initial negative biopsy tissues were significantly associated with a positive repeat biopsy outcome of PCa (Table S6, Figure 2 B and C). In addition, the immunoreactivity of p‐STAT3 and Mcm2 + luminal to basal ratio were significantly associated with a positive repeat biopsy outcome of HGPCa (Table S5, Figure 2 B and C).

To further confirm the field effects of p‐STAT3, Mcm2 + luminal to basal ratio, and MSR1 + cell number, we detected the immunoreactivity of the p‐STAT3 and MSR1 + cell number in the PCa, pericancer tissues near and far from tumor core, and benign tissues. We also measure the Mcm2 + luminal to basal ratio in pericancer tissues near and far from tumor core, and benign tissues. Figure 2D showed that the immunoreactivity of the biomarkers significantly differed in the PCa sections and/or pericancer tissues compared to the benign prostate sections. In addition, in the pericancer tissues, the difference could still be observed as far as 10 mm from the tumor area as compared with the benign prostate tissues (Figure 2D).

Based on above results, multivariate analysis was performed by stepwise logistic regression modeling to construct a model using all factors that were significant in the univariate analysis (*P* < .1, Table S6). Finally p‐STAT3, Mcm2, and MSR1 were used to construct the model for predicting cancers in the repeat biopsy cohort. When predicting HGPCa in the repeat biopsy cohort, p‐STAT3 and Mcm2 were selected to construct the model (Table S7). The multivariate analysis results and coefficients for these biomarkers in models predicting cancers and HGPCa were shown in Table S7. We defined the biomarker index as the model's prediction probability multiplied by 100 (ranging from 0 to 100). So biomarker index was calculated as 100 × 1/(e^‐P^ + 1). e is natural logarithm and when predicting cancers, P = p‐STAT3 × 0.009138 + MSR1 × −0.005494 + Mcm2 × 0.133981 + (−0.654652). When predicting HGPCa, P = p‐STAT3 × 0.011919 + Mcm2 × 0.147794 + (−3.966242). Biomarker index represents the joint efforts of the selected biomarkers.

### 3.2 Validation of biomarker index

**Figure 3:**
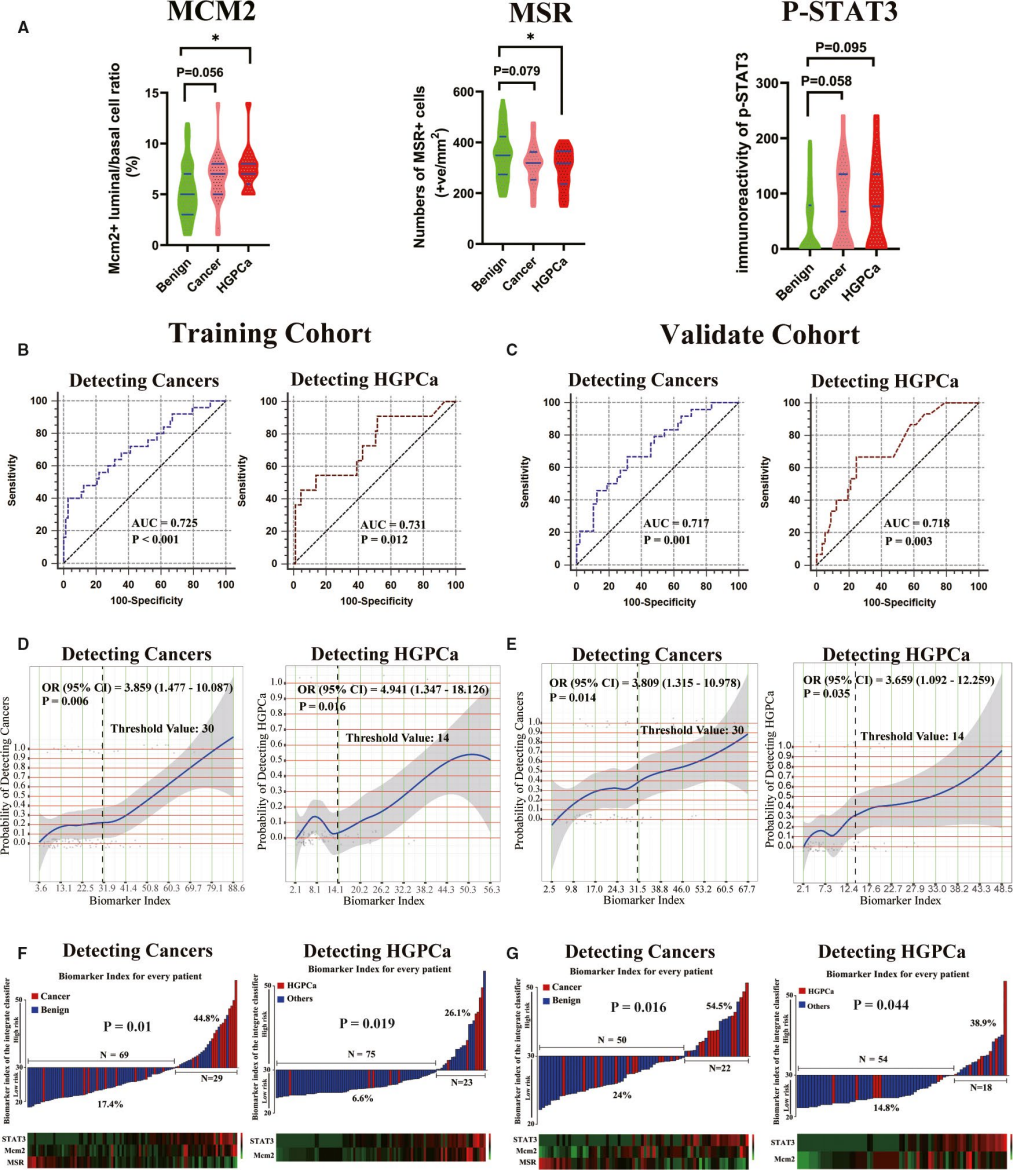
Validation of biomarker index. (A) Differential immunoreactivity of p‐STAT3, Mcm2 + luminal to basal ratio and MSR + cell number in the initial negative biopsy tissues of the validation cohort grouped by initial biopsy result. (B, C) ROC curve of biomarker index when predicting cancer and HGPCa in the training (B) and validation (C) cohorts. (D, E) LOESS smooth curves showed the probability of detecting cancer or HGPCa increased with the increase of the biomarker index in the training (D) and validation (E) cohorts. The corresponding biomarker index value of the inflection region of curves was selected as threshold values. According to the LOESS curves, 30 and 14 were selected as threshold values to divide patients into high‐risk and low‐risk groups when predicting cancer and HGPCa respectively. The corresponding OR (odds ratio) and 95% CI were calculated by univariate logistic regression according to the threshold values. (F, G) PCa or HGPCa detection rate in the high‐risk and low‐risk patients in the training (F) and validation (G) cohorts

The differential immunoreactivity of the three biomarkers in the initial negative biopsy tissues of the benign, PCa, and HGPCa groups from the validation cohort were shown in Figure 3A. We constructed the biomarker index in the validation cohort according to the biomarkers and corresponding coefficients from the training cohort. To evaluate the predictive value of the biomarker index as a linear variable in the training and validation cohorts, ROC curve was performed. The AUC of the biomarker index was 0.725 and 0.731, respectively, when predicting the positive biopsy results and HGPCa in the training cohort and was 0.717 and 0.718, respectively, in the validation cohort (Figure 3B and 3C). Then, LOESS smooth curves of the biomarker index for predicting cancer and HGPCa in the training and validation cohorts are shown in Figure 3D and E. Finally, we generated threshold values according to the LOESS smooth curves (Figure 3D and E) and divided patients into high‐risk and low‐risk groups. And univariate analysis showed that our grouping strategy was strongly correlated with repeat biopsy results (Figure 3D and E). Also Figure 3F and 3G showed high‐risk patients had a significantly higher probability of PCa and HGPCa compared with low‐risk patients in both the training and validation cohorts.

### 3.3 The role of biomarker index in repeat biopsy decision‐making processes

Clinicopathological characteristics of the training cohort are shown in Table 1. In total, 25 (25.5%) of 98 patients had a positive biopsy after a previous negative biopsy during the follow‐up. The majority of cancers diagnosed were GG 1 (n = 14; 56.0%), whereas 11 (44.0%) were HGPCas (2 cases with GG2, 6 cases with GG3, and 3 cases with GG 4 or higher).

The univariate logistic analysis showed that the biomarker index, fPSA% and serum PSA were predictive factors associated with a positive repeat biopsy outcome (Table 1). The AUC of each variable when discriminating between patients with and without positive biopsy is given in Table 2. The biomarker index possessed the maximum AUC (0.72), followed by fPSA% (0.65), ASAP history (0.60), and serum PSA (0.58).

**Table 2:** Multivariate logistic analysis with corresponding predictive accuracy for each variable

Variables	Biopsy outcome of prostate cancer			Biopsy outcome of HGPCa		
	OR (95%CI)	*P* Value	AUC	OR (95%CI)	*P* Value	AUC
Age (Continuous)	1.04 (0.95‐1.14)	.39	0.52	0.97 (0.85‐1.10)	.63	0.55
f/t PSA ratio (Continuous)	0.88 (0.79‐0.99)	.031	0.65	0.85 (0.73‐0.99)	.039	0.66
Serum PSA (Continuous)	1.11 (0.98‐1.26)	.098	0.58	1.19 (1.01‐1.40)	.043	0.62
DRE, n, (Abnormal)	3.11 (0.35‐27.85)	.31	0.51	1.31 (0.071‐24.45)	.86	0.51
ASAP history (Present)	7.10 (1.31‐38.57)	.023	0.60	2.31 (0.14‐37.84)	.56	0.53
HGPIN history (Present)	2.62 (0.37‐18.53)	.34	0.52	5.14 (0.28‐96.13)	.27	0.52
Biomarker index	1.06 (1.03‐1.09)	<.001	0.72	1.08 (1.03‐1.13)	.002	0.73

Abbreviations: AUC: Area under curve;CI: Confidence interval; HGPCa: High grade prostate cancer; OR: odd ratio.

When predicting HGPCa, the univariate analysis showed the biomarker index, fPSA%, and serum PSA were associated with HGPCa in the repeat biopsy. Table 2 shows that the biomarker index had the maximum AUC (0.73), followed by fPSA% (0.66) and serum PSA (0.62).

In the multivariate logistic analysis, the fPSA%, ASAP history and biomarker index were independent predictive factors associated with cancers in the repeat biopsy. However, when predicting HGPCa, the fPSA%, serum PSA, and biomarker index were independent predicting factors (Table 2).

Various models were constructed using multivariate logistic regression analysis. The ROC was used to evaluate the value of each model for predicting cancer or HGPCa in the repeat biopsy (Table 3). The base model incorporating factors of age, fPSA%, serum PSA, DRE, and ASAP/HGPIN history, had an AUC of 0.722 and 0.735, respectively, when predicting cancer and HGPCa (Table 3). In addition, the addition of the biomarker index (full model) improved the AUC of the base model for predicting cancer (0.842) and HGPCa (0.842) in the repeat biopsy (Table 3). Then, the calibration plots were drawn to internally validate each model (Figure S2 A and S1B). The calibration plots of both the base and full models closely paralleled the ideal prediction line and the departures from ideal predictions were within acceptable scope when observing the rate of cancer or HGPCa (Figure S2A and S1B). Finally, a nomogram was constructed incorporating predictors likely to be associated with detecting cancer or HGPCa (*P* < .1 in the multivariate logistic regression analysis, Table 2) and is shown in Figure S2C, and D

**Table 3:** ROC curves of each model for predicting the risk of any prostate cancer or HGPCa at re‐biopsy

	Biopsy outcome of any prostate cancer		Biopsy outcome of high‐grade prostate cancer	
	AUC	Gain in AUC	AUC	Gain in AUC
Base model	0.722		0.735	
Full model	0.842	0.120	0.842	0.113

Abbreviations: AUC, area under curve; ROC, receiver operating characteristic.

Base model: Age, PSA, %fPSA, DRE, ASAP, and HGPIN.

Full model: Base model + Biomarker index.

**Figure 4:**
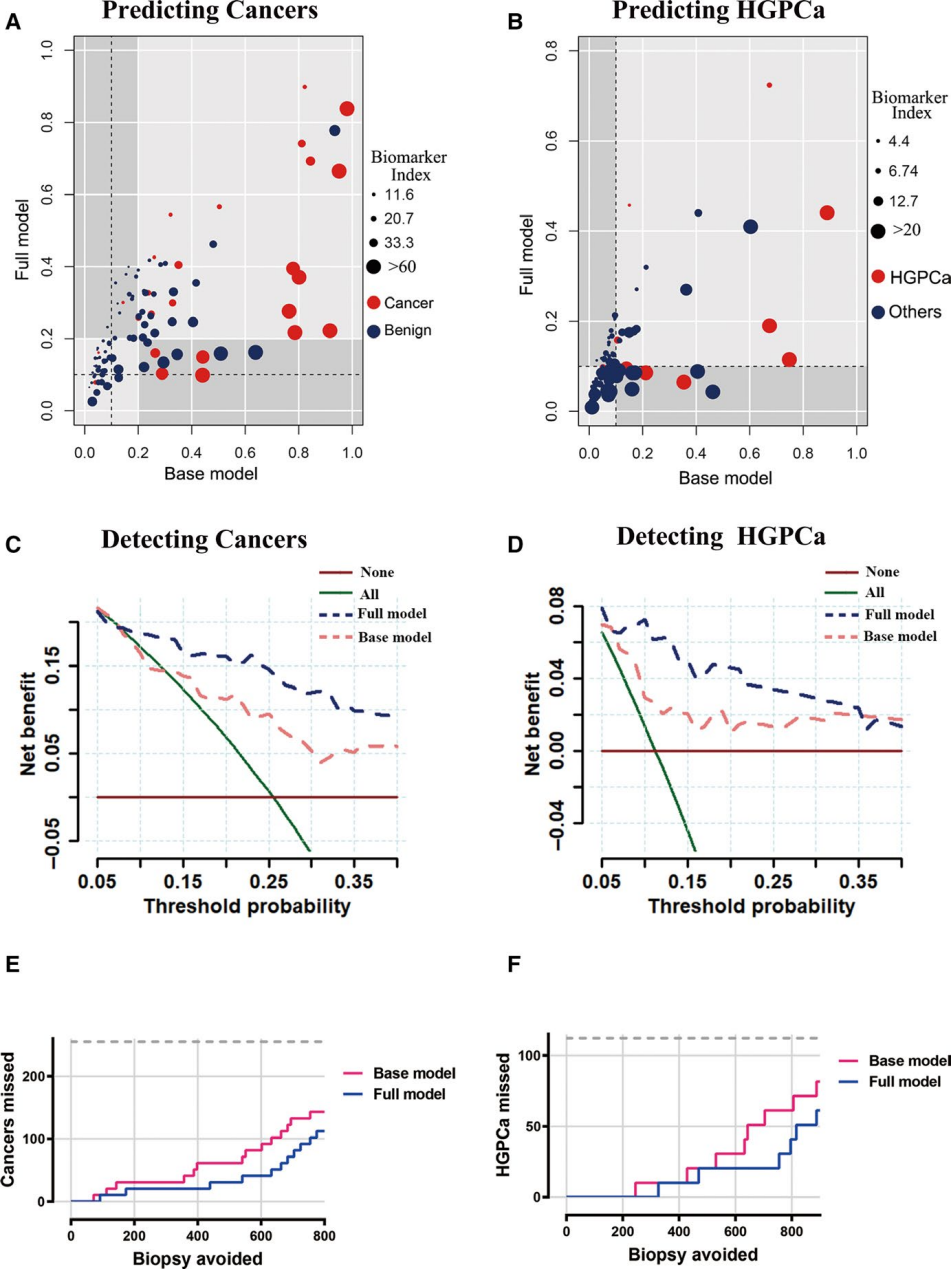
Role of biomarker index in repeat biopsy decision‐making process. (A, B) Reclassification of base model score categories by biomarker‐clinicopathological classifiers (full model) score for patients in the cohort. Based on LOESS curves of models’ scores, 20 and 11 were selected as threshold value to reclassify patients into high‐risk and low‐risk groups when predicting cancers (A) and HGPCa (B). Individual patients were represented as dots colored by repeat biopsy outcomes; sizes of dots represented the biomarker index as indicated. Gray quadrants represented situations in which the full model classifier reclassifies patients compared to the base model. Patients who did not have cancer or HGPCa (blue dots) in the bottom‐right quadrant and patients who had cancer or HGPCa (red dots) in the top‐left quadrant were reclassified correctly by the full model. (C, D) Decision curves for outcome of cancer (C) and HGPCa (D) using the base model and full model. Strategies for biopsies in all men (biopsy all) or no men (biopsy none) were also shown. The line with the highest net benefit at any particular threshold probability for biopsy (x‐axis) will yield the best clinical results. E, F: Number of biopsies that could be avoided and number of cancers (E) or HGPCa (F) that could be missed per 1000 patients based on prediction models at different predicted probabilities

Patients were risk‐stratified according to their base and full model scores into high‐risk and low‐risk groups when predicting cancers (threshold value: 0.2) and HGPCas (threshold value: 0.11). The threshold values were defined by LOESS curve and showed in Figure S3A and B. The categorization by the “clinical‐only” base model was compared with the full model (biomarker‐clinicopathological classifiers). Figure 4A showed that, when predicting cancers, the full model reclassified 24 patients into different predicted risk categories, of which 18 (74.0%) were correctly reclassified. Similarly, when predicting HGPCa, the full model reclassified 28 patients originally risk stratified by base model, of which 21 (75.0%) were correctly reclassified (Figure 4B).

The decision curve analysis indicated that in the range of threshold probabilities from 10% to 40%, the full model offered net benefit over the base model in predicting either cancer (Figure 4C) or HGPCa (Figure 4D).

To put these results in a clinical context, we expanded our repeat biopsy cohort to 1,000 people proportionally and calculated the number of biopsies and number of cancers or HGPCas that could be avoided or missed per 1000 patients based on prediction models at different predicted probabilities (Table 4). For example, we considered the scenario in which a clinician would recommend a biopsy to patients with a predicted probability of 10%. Applying this rule, the full model would reduce the number of biopsies by 34.7% while delaying the diagnosis of 7.8% cancers per 1000 men with suspicion of cancer, and when detecting HGPCa, the full model would reduce the number of biopsies by 73.5% while delaying the diagnosis of 20 HGPCas per 1000 men with suspicion of HGPCa. Compared with the base model, Figure 4 E and F showed that the full model appeared to overlook fewer cancers (Figure 4E) and HGPCas (Figure 4F) while avoiding the same number of biopsies.

**Table 4:** Number of biopsies that could be avoided for repeat biopsy at 5%, 10%, 15%, 20% threshold

	Any cancer				HGPCa			
	Biopsies		Cancer		Biopsies		Cancer	
	Performed	Avoided	Found	Missed	Performed	Avoided	Found	Missed
Biopsy all	1000	0	255	0	1000	0	112	0
Full model								
>5%	857(769‐ 917)	143(83‐231)	245(198‐254)	10(1‐57)	541(437‐641)	459(359‐563)	102(64‐111)	10(1‐48)
>10%	653(549‐745)	347(255‐451)	235(185‐251)	20(4‐70)	265(184‐366)	735(634‐816)	92(53‐108)	20(4‐59)
>15%	561(457‐660)	439(340‐543)	224(173‐247)	31(8‐83)	194 (124‐289)	806(711‐876)	71(35‐98)	41(14‐80)
>20%	479(378‐582)	520(418‐622)	224(173‐247)	31(8‐83)	122(68‐208)	878(792‐932)	61(28‐92)	51(20‐84)

Abbreviations: HGPCa, High‐grade prostate cancer.

## 4 DISCUSSION

Relatively higher rates of PCa on repeat biopsy, ranging from 20% to 34%, have been reported by previous studies.^3^, ^4^, ^5^ However, the criteria or indications for making a decision to perform a repeat biopsy procedure are not well defined. The AUC of models constructed by traditional clinical parameters in the repeat biopsy is only approximately 70%.^5^ In our study, the AUC of the baseline model was only 0.722 and 0.735 when predicting cancer and HGPCa, respectively. Therefore, it is necessary to identify novel biomarkers to help urologists in the decision‐making processes.

Over the past decades, to improve the diagnosis accuracy of PCa many attempts has been made. A novel urine exosome gene expression assay has been reported by a large prospectively study. Plus this assay with traditional clinical parameters could significant improved discrimination between HGPCa and others at initial biopsy.^30^, ^31^ Other biomarkers like urinary PCA3 and TMPRSS2:ERG,^32^ and IsoPSA™ (a blood based, structure focused assay)^33^ can improve the diagnostic accuracy of traditional clinical parameters and showed promising predicting ability at initial biopsy.

However unlike the initial biopsy, the previous negative biopsy tissue may provide valuable information for repeat biopsy patients.. In our study, after screening a group of biomarkers which may have potential field effects, we found that the immunoreactivity of p‐STAT3, Mcm2 + and/or MSR1 were associated with cancer and HGPCa at repeat biopsy. The field effects of those biomarkers were further confirmed in our analysis. The biomarker index that combined the effects of the biomarkers showed promising discrimination abilities both in the training and validation cohorts. The AUC of the biomarker index when discriminating cancer or HGPCa from a benign prostate was 0.725 and 0.731, respectively, which was higher than any other single traditional parameter and any single biomarker alone. More importantly, the full model supplemented with the biomarker index with traditional parameters both had an AUC0.842 when predicting cancer and HGPCa in the repeat biopsy (Table 3), which significantly improved the AUC of the base model. In the decision‐making analysis, we found that the full model could offer more net benefits than other models, which could help urologists to make better decisions.

Disorders in DNA replication and cell proliferation are basic aspects of many cancers, including PCa. Mcm2, a protein involved in the eukaryotic DNA replication procedure and that serves as the convergence point of several cell proliferation pathways, could be a promising biomarker that can provide relevant diagnostic and prognostic information.^34^, ^35^, ^36^ From normal to tumor tissues, a shift in Mcm2 immunoreactivity from the basal to luminal cell compartment was noted by a previous study.^11^ Moreover the Mcm2 luminal to basal cell ratios were significantly higher in the normal glands from prostates with cancer than in the normal glands from prostates free of cancer.^11^ In our study, we found that the Mcm2 luminal to basal cell ratios not only showed considerable filed effects but also were significantly associated with positive repeat prostate biopsy results. Also in a prospective study, detection of MCM2 in voided urinary samples could serve as a promising biomarker for diagnosis of bladder cancers.^37^

Previous studies have demonstrated that many inflammatory and immune cells contribute to carcinogenesis or progression of PCa, including MSR1 + cells.^38^, ^39^, ^40^, ^41^ Decreased infiltration of MSR1 + cells has been proven to be associated with the progression of PCa and worse clinical outcomes.^41^ Norio et al reported that the number of MSR1 + cells in previous negative biopsy specimens was significantly lower in patients with cancer than those without cancer at repeat biopsy, which indicated that the MSR1 + cells count in the initial negative biopsy might be a promising biomarker in predicting a positive repeat biopsy outcome.^14^ However, the study failed to evaluate the clinical effects of MSR1 in a repeat biopsy cohort. In our study, we found that MSR1 + cell count was significantly associated with cancers in the repeat biopsy.

STAT3 is a member of the JAK‐STAT signaling pathway, and constitutively activated STAT3 is correlated with the prognosis, progression and metastasis of various cancers including PCa.^42^, ^43^, ^44^ Dhir et al detected significantly higher levels of constitutive STAT3 activity in both prostate carcinomas and pericancerous glands compared to the normal prostates without the influence of PCa.^19^ Moreover another study and our study showed that the immunoreactivity of p‐STAT3 in the initial negative biopsy tissues could be used to predict the positive biopsy outcome.^18^ However, the diagnosis role of p‐STAT3 in other cancers were sparely reported.

There are several limitations to this study. First, this study was a retrospective study. Second, many variables, such as prostate volume, PSA velocity, and family history, were not included in our study due to limited sample size. An additional limitation of this study would be that lack of further external validation. We believe that future large sample, multicenter studies incorporating results from more variables or biomarkers would provide a more profound understanding of the repeat biopsy.

Taken together, the biomarker index constructed by p‐STAT3, Mcm2 luminal to basal ratio and/or MSR1 + cell count is significantly associated with positive repeat prostate biopsy results, and supplementing this biomarker index with traditional clinical parameters may help urologists in decision‐making processes regarding who will benefit from repeat biopsy.

## Conflict of interest

The authors have no conflict of interest.

## AUTHOR CONTRIBUTION

LY, LX, WL, and WZ designed study. LX, WL, ZX, HX, JH, and ZL analyzed data. LY and LX reviewed the manuscript. LX and WL wrote the manuscript. YK and WL performed immunohistochemistry research and analysis. CY, YC, WZ, and ZX collected data.

## Supporting information

Fig S1

Fig S2

Fig S3

Table S1

Table S2

Table S3

Table S4

Table S5

Table S6

Table S7

## Data Availability

Data sharing is not applicable to this article as no new data were created or analyzed in this study.
