# Supplementary figures and images for: Biomarkers in previous histologically negative prostate biopsies can be helpful in repeat biopsy decision‐making processes
Source: Cancer Med. 2020 Aug 28;9(20):7524–36. doi: 10.1002/cam4.3419 (PMC7571822; doi:10.1002/cam4.3419)

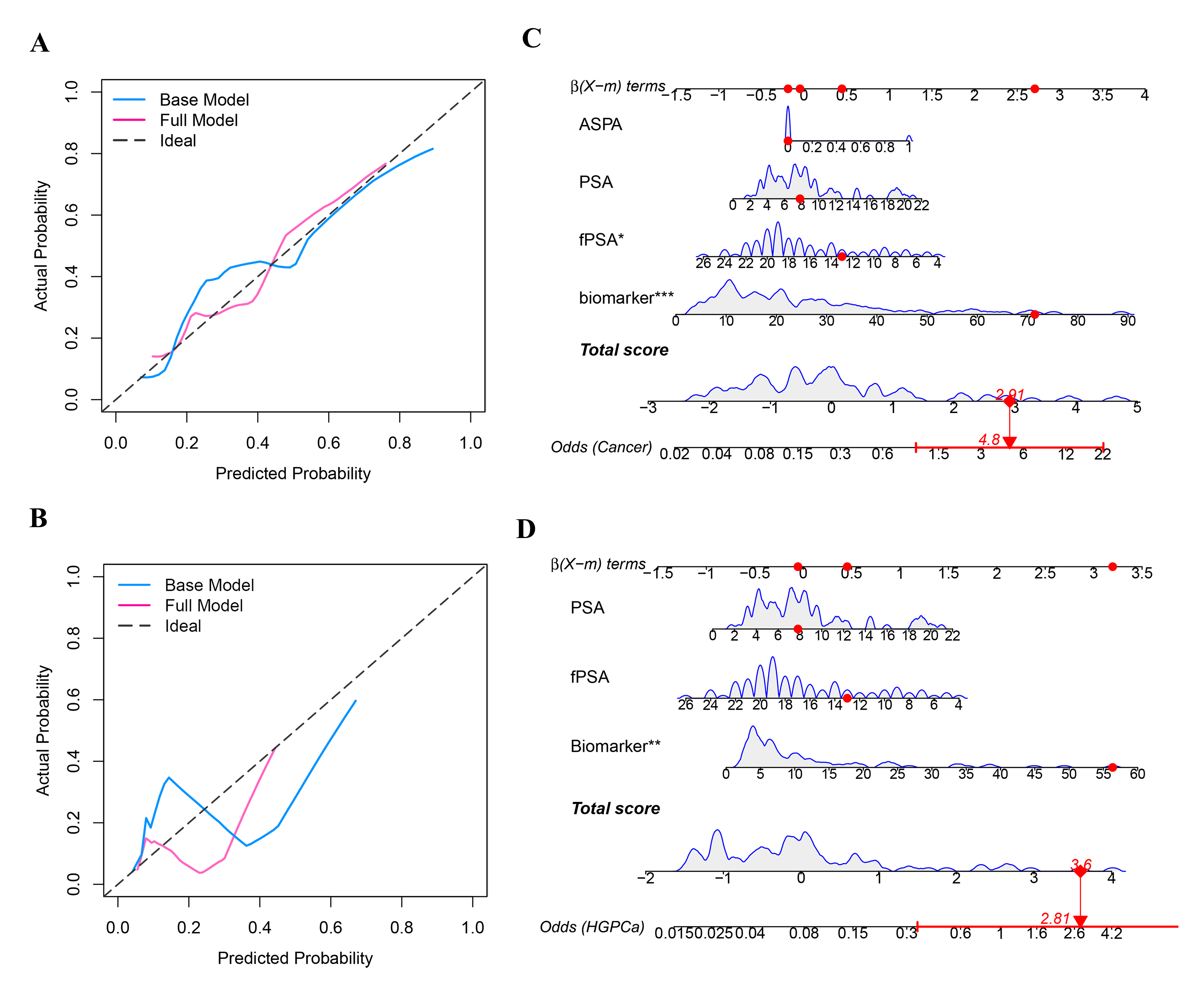

Supplement: Supplementary file 2 — Fig S2 [file CAM4-9-7524-s002.tif]

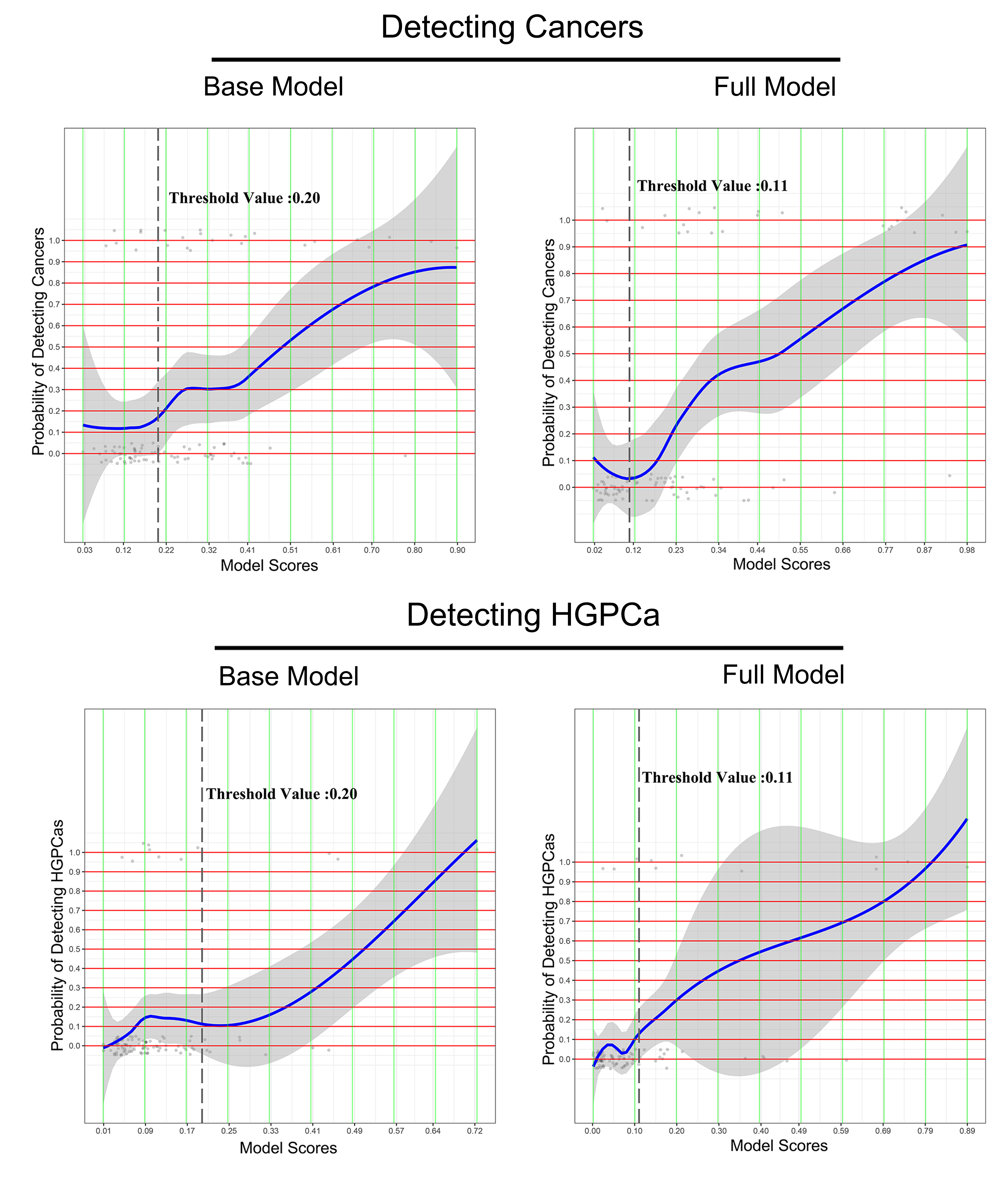

Supplement: Supplementary file 3 — Fig S3 [file CAM4-9-7524-s003.tif]
