# Supplementary material for: Biomarkers in previous histologically negative prostate biopsies can be helpful in repeat biopsy decision‐making processes
Source: Cancer Med. 2020 Aug 28;9(20):7524–36. doi: 10.1002/cam4.3419 (PMC7571822; doi:10.1002/cam4.3419)
Supplement: Supplementary file 4 — Table S1 [file CAM4-9-7524-s004.docx]

| Supplementary Table S1: Clinical characteristics of the initial biopsy cohort (the validation cohort). | | | | |
| --- | --- | --- | --- | --- |
| Variables | Total | Initial Biopsy Results | | |
| No. of Pt | 72 | Benign (n=48) | Any prostate cancer (n=24) | High grade prostate cancer (n=15) |
| Age, yr, (mean ± SD) | 67.61±6.14 | 66.96±6.53 | 68.92±5.13 | 69.13±5.88 |
| f/t PSA ratio, %, (mean ± SD) | 16.32±5.49 | 17.96±5.31 | 13.05±4.30 | 11.58±3.75 |
| Clinical serum PSA, ng/mL, (mean ± SD) | 8.17±5.0 | 6.22±2.60 | 12.06±6.32 | 14.57±6.49 |
| No. of suspicious DRE, n, (%) | 19(26.39) | 6(12.5) | 13(54.17) | 10(66.67) |
| No. of biopsy cores (mean ± SD), (mean ± SD) | 7.35±1.94 | 7.19±2.02 | 7.67±1.76 | 7.80±1.90 |
| No. of positive cores, (mean ± SD) | \ | \ | 3.08±1.14 | 3.4±1.06 |
