# Supplementary material for: Biomarkers in previous histologically negative prostate biopsies can be helpful in repeat biopsy decision‐making processes
Source: Cancer Med. 2020 Aug 28;9(20):7524–36. doi: 10.1002/cam4.3419 (PMC7571822; doi:10.1002/cam4.3419)
Supplement: Supplementary file 5 — Table S2 [file CAM4-9-7524-s005.docx]

| Supplementary Table S2: Clinical Characteristics of the PCa patients | | | |
| --- | --- | --- | --- |
| Patients | Age (median, range) | Sources | Pathological grade |
|  |  |  |  |
| PCa (n=16) | 68(43-77) | Radical prostatectomy | Gleason 6: n=3 |
|  |  |  | Gleason 7: n=7 |
|  |  |  | Gleason 8: n=4 |
|  |  |  | Gleason 9: n=2 |
