# Supplementary material for: Biomarkers in previous histologically negative prostate biopsies can be helpful in repeat biopsy decision‐making processes
Source: Cancer Med. 2020 Aug 28;9(20):7524–36. doi: 10.1002/cam4.3419 (PMC7571822; doi:10.1002/cam4.3419)
Supplement: Supplementary file 6 — Table S3 [file CAM4-9-7524-s006.docx]

| Supplementary Table S3: Clinical Characteristics of the benign prostate patients | | | |
| --- | --- | --- | --- |
| Patients | Age (median, range) | Sources |  |
| Normal prostate  (n=16) | 64(57-75) | Radical cystectomy |  |
|  |  |  |  |
