# Supplementary material for: Biomarkers in previous histologically negative prostate biopsies can be helpful in repeat biopsy decision‐making processes
Source: Cancer Med. 2020 Aug 28;9(20):7524–36. doi: 10.1002/cam4.3419 (PMC7571822; doi:10.1002/cam4.3419)
Supplement: Supplementary file 7 — Table S4 [file CAM4-9-7524-s007.docx]

| Supplementary Table S4. Antibodies Used | | | |
| --- | --- | --- | --- |
| Antibody | Item number | Dilution | Company |
| Anti-CD3 | 17617-1-AP | 1:200 | Proteintech Group, Inc. Wuhan, China |
| Anti-CD68 | ab213363 | 1:800 | Abcam. Cambridge, MA, USA |
| Anti-Ki-67 | ARG53222 | 1:200 | Arigo biolaboratories Corp. Shanghai, China |
| Anti-Mcm-2 | ab108935 | 1:200 | Abcam. Cambridge, MA, USA |
| Anti-activated caspase-3 | ab2302 | 1:100 | Abcam. Cambridge, MA, USA |
| Anti-VEGF | 19003-1-AP | 1:200 | Proteintech Group, Inc. Wuhan, China |
| Anti-CD31 | 11265-1-AP | 1:800 | Proteintech Group, Inc. Wuhan, China |
| Anti-p-STAT3 | 9145 | 1:200 | Cell Signaling Technology. Boston, MA, USA |
| Anti-P-AKT | ab38449 | 1:200 | Abcam. Cambridge. MA, USA |
| Anti-MSR | ab217843 | 1:200 | Abcam. Cambridge. MA, USA |
