# Supplementary material for: Biomarkers in previous histologically negative prostate biopsies can be helpful in repeat biopsy decision‐making processes
Source: Cancer Med. 2020 Aug 28;9(20):7524–36. doi: 10.1002/cam4.3419 (PMC7571822; doi:10.1002/cam4.3419)
Supplement: Supplementary file 8 — Table S5 [file CAM4-9-7524-s008.docx]

| Supplementary Table S5: H-Score of biomarkers stratiﬁed by biopsy results in the training cohort | | | | | | | |
| --- | --- | --- | --- | --- | --- | --- | --- |
| Biomarkers, (Median, quartile range) | Total (n=98) | Repeat Biopsy Results | | |  | p Value | |
|  |  | Benign (n=73) | Any prostate cancer (n=25) | HGPCa (n=11) |  | Any cancer vs Benign | HGPCa vs Benign |
| P-STAT3 | 49.00 (0-86.75) | 44.00 (0-76.50) | 64.00 (0-137.00) | 97.00 (0-142.00) |  | 0.002 | 0.003 |
| MSR1 | 342.33 (290.12-404.10) | 362.66 (300.49-419.64) | 320.00(230.57-361.00) | 320.00 (235.00-370.00) |  | 0.013 | 0.033 |
| MCM2 | 6.00 (4.00-8.00) | 6.00 (4.00-6.50) | 7.00 (4.50-9.50) | 7.00 (4.00-14.00) |  | 0.028 | 0.015 |
| CD31 | 12.00 (7.00-18.00) | 11.00 (7.00-17.00) | 13.00 (7.00-20.50) | 13.00 (10.00-18.00) |  | 0.247 | 0.412 |
| Ki-67 | 15.00 (10.00-18.00) | 15.00 (10.00-17.00) | 15.00 (11.00-19.00) | 17.00 (10.00-19.00) |  | 0.238 | 0.359 |
| CD3 | 174.00 (136.00-205.00) | 165.00 (140.00-199.50) | 182.00 (95.50-240.50) | 182.00 (104.00-258.00) |  | 0.810 | 0.657 |
| CD68 | 189.00 (152.50-264.00) | 189.00 (151.00-264.50) | 174.00(150.50-256.00) | 167.00 (138.00-258.00) |  | 0.707 | 0.423 |
| a-Casp3 | 85.84(0-118.40) | 88.80 (0-115.44) | 82.88 (0-118.40) | 82.88 (0-118.40) |  | 0.925 | 0.830 |
| VEGF | 77.00 (0-119.85) | 74.00 (0-118.40) | 88.80 (0-172.00) | 111.00 (0-200.00) |  | 0.149 | 0.109 |
| P-AKT | 133.50 (0-186.25) | 132.00 (0-175.00) | 157.00 (0-229.50) | 187.00 (0-217.00) |  | 0.606 | 0.552 |
| HGPCa=High grade prostate cancer | | | | | | |  |
