# Supplementary material for: Biomarkers in previous histologically negative prostate biopsies can be helpful in repeat biopsy decision‐making processes
Source: Cancer Med. 2020 Aug 28;9(20):7524–36. doi: 10.1002/cam4.3419 (PMC7571822; doi:10.1002/cam4.3419)
Supplement: Supplementary file 9 — Table S6 [file CAM4-9-7524-s009.docx]

| Supplementary Table S6: Univariable logistic Regression Analysis with corresponding predictive accuracy for biomarkers | | | | | | | |
| --- | --- | --- | --- | --- | --- | --- | --- |
| Variables | Biopsy outcome of any prostate cancer | | |  | Biopsy outcome of HGPCa | | |
|  | OR (95% CI) | P Value | AUC |  | OR (95% CI) | P Value | AUC |
| P-STAT3 | 1.013(1.004～1.022) | 0.005 | 0.64 |  | 1.013(1.002～1.024) | 0.017 | 0.659 |
| MSR | 0.994(0.989～0.999) | 0.017 | 0.658 |  | 0.993(0.986～1.000) | 0.06 | 0.65 |
| MCM2 | 1.136(1.009～1.278) | 0.035 | 0.616 |  | 1.166(1.012～1.344) | 0.033 | 0.624 |
| CD31 | 1.041(0.973～1.113) | 0.246 | 0.567 |  | 1.030(0.940～1.129) | 0.523 | 0.582 |
| Ki-67 | 1.055(0.965～1.153) | 0.237 | 0.592 |  | 1.051(0.931～1.187) | 0.42 | 0.59 |
| CD3 | 1.001(0.994～1.008) | 0.808 | 0.555 |  | 1.002(0.992～1.012) | 0.66 | 0.556 |
| CD68 | 0.999(0.993～1.005) | 0.704 | 0.538 |  | 0.996(0.987～1.005) | 0.403 | 0.59 |
| a-Casp3 | 1.000(0.993～1.008) | 0.924 | 0.505 |  | 1.001(0.991～1.011) | 0.827 | 0.513 |
| VEGF | 1.005(0.998～1.012) | 0.15 | 0.58 |  | 1.007(0.998～1.016) | 0.147 | 0.612 |
| P-AKT | 1.001(0.996～1.006) | 0.602 | 0.549 |  | 1.002(0.995～1.009) | 0.596 | 0.574 |
| Biomarker index | 1.117(1.053～1.185) | <0.001 | 0.725 |  | 1.207(1.074～1.357) | 0.002 | 0.731 |

HGPCa:High-grade prostate cancer; AUC: Area under curve; OR: Odd ratio; CI: Confidence interval.
