# Supplementary material for: Biomarkers in previous histologically negative prostate biopsies can be helpful in repeat biopsy decision‐making processes
Source: Cancer Med. 2020 Aug 28;9(20):7524–36. doi: 10.1002/cam4.3419 (PMC7571822; doi:10.1002/cam4.3419)
Supplement: Supplementary file 10 — Table S7 [file CAM4-9-7524-s010.docx]

| Supplementary Table S7: Multivariate stepwise logistic regression analysis with corresponding coefﬁcients for biomarkers | | | | | | | | | | |
| --- | --- | --- | --- | --- | --- | --- | --- | --- | --- | --- |
| Variables | Biopsy outcome of any prostate cancer | | | | |  | Biopsy outcome of HGPCa | | | |
|  | OR (95% CI) | P Value | Logistic coefficient |  | OR (95% CI) | | | P Value | Logistic coefficient |  |
| P-STAT3 | 1.009(1.000～1.018) | 0.050 | 0.009138 |  | 1.012(1.001～1.023) | | | 0.028 | 0.011919 |  |
| MSR | 0.995(0.989～1.000) | 0.049 | -0.005494 |  | / | | | / | / |  |
| MCM2 | 1.143(1.000～1.307) | 0.049 | 0.133981 |  | 1.159(0.996～1.350) | | | 0.057 | 0.147794 |  |

HGPCa: High grade prostate cancer; AUC: Area under curve; OR: Odd ratio; CI: Confidence interval.
